# Supplementary material for: Differential and Synergistic Effects of Low Birth Weight and Western Diet on Skeletal Muscle Vasculature, Mitochondrial Lipid Metabolism and Insulin Signaling in Male Guinea Pigs
Source: Nutrients. 2021 Nov 29;13(12):4315. doi: 10.3390/nu13124315 (PMC8704817; doi:10.3390/nu13124315)
Supplement: Supplementary file 1 [file nutrients-13-04315-s001.zip › nutrients-1440930-supplementary.pdf]

Supplementary Tables

Supplementary Table S1. Composition of the experimental control diet (CD) and Western diet (WD)

Item	CD* (TD.110240)	WD* (TD.110239)
Main ingredients (g kg−1)		
Isolated soy protein	210	255
L-METHIONINE	2.47	3
Sucrose	100	190
Fructose	—	65
Corn starch	354	—
Maltodextrin	93	93
Cellulose	130	130
Soybean oil	60	—
Cocoa butter	—	50
Lard	—	55
Coconut oil	—	95
Cholesterol	—	2.5
Vitamin Mix Teklad (40060)	10	12.3
Vitamin C, l-ascorbyl-2-polyphosphate (35%)	0.61	0.75
Folic acid	0.008	0.01
Calcium phosphate dibasic	17.66	21.5
Potassium citrate, monohydrate	9.85	12
Magnesium oxide	2.63	3.2
Potassium chloride	3.29	4
Sodium chloride	1.64	2
Calcium carbonate	4.1	5
Ferric citrate	0.33	0.4
Manganese sulfate, monohydrate	0.164	0.2
Zinc carbonate	0.05	0.06
Cupric sulfate	0.0164	0.02
Potassium iodate	0.0008	0.001
Chromium potassium sulfate, dodecahydrate	0.008	0.01
Sodium selenite, pentahydrate	0.0008	0.001
Ammonium paramolybdate, tetrahydrate	0.0002	0.0003
Chemical composition		
Protein (% kcal)	21.6	21.4
Fat (% kcal)	18.4	45.3
Carbohydrates (% kcal)	60	33.3
Energy (kcal g−1)	3.4	4.2
Fatty acid composition (% of total fatty acids)		
Lauric acid (C12:0)	—	23.12
Myristic acid (C14:0)	—	8.83
Palmitic acid (C16:0)	11	17.32
Stearic acid (C18:0)	4	13.24
Oleic acid (C18:1*cis*9)	23.5	24.4
Linoleic acid (C18:2*n*−6)	53.4	4.2
α-Linoleic acid (C18:3*n*−3)	8	0.03

*Diets were formulated in the Harlan Laboratories (Madison, WI, USA)

Supplementary Table S2. Primers used for analysis of gene expression by qRT-PCR

Gene	Accession No.	Anneal Temp (°C)	Forward Sequence (5'-3')	Reverse Sequence (3'-5')	Efficiency
VLCAD	XM_003466 183.2	59	CAAACTGGCAGTG ACGGCT	TTGGTGGGGGTCAGA CTGTA	91.2%
MCAD	XM_003479 087.2	59	CGAGTTGACCGAA CAGCAGA	CAACAGGCATTTGCC CCAAG	94.4%
KT	XM_003464 099.2	59	TAAGGTCCTACGC AGTGGTTG	CTCCATAAGCCCTCT TCCCAC	90.8%
PDK4	XM_003475 111.2	59	GCAGTGGTCCAAG ATGCCTT	TGGTGTTCAACTGTT GCCCT	94.2%
CPT1b	XM_003461 559.1	59	AGCTCCCCATTCC TAGCAGA	CGCTGAGCATTCGTC TCTGA	96.7%
PGC1α	XM_003467 408.2	56	CAAGACCAGTGAA TTGAGGG	CATCCTTTGGGGTCTT TGAG	92.5%
PPARα	NM_001173 004.1	56	AGATCCAGAAAAA GAACCGC	TTTTGCTTTCTCAGAC CTCG	91.8%
SIRT1	XM_005005 505.1	58	TTGCAACTGCATC TTGCCTG	TCATGGGGTATGGAA CTTGGAA	103.8%
SIRT3	XM_004999 541.1	58	CATGGCGGATCTG CTACTCA	AGGCTGCATGTTGTG GTTTG	93.8%
β-actin	NM_001172 909.1	59	AAGAGATGTGGCC TCAAAGC	CAGGAACAGGCCGTA GAGTG	100.6%

Supplementary Table S3. Specifications and catalog numbers of antibodies used for immunoblotting

Protein	Species	Dilution	Blocking	Company	Catalogue

			Solution		No.
IRβ	Rabbit Monoclonal	1:1000	5% BSA	Cell Signaling Technology	3025
pIRβ (Tyr 1150/1151)	Rabbit Monoclonal	1:1000	5% BSA	Cell Signaling Technology	3024
pAKT (Ser 473)	Rabbit Monoclonal	1:1000	5% BSA	Cell Signaling Technology	4060
pAKT (Thr308)	Rabbit Monoclonal	1:1000	5% BSA	Cell Signaling Technology	2965
PKCθ	Rabbit Polyclonal	1:1000	5% BSA	Cell Signaling Technology	2059
PKCε	Rabbit Monoclonal	1:1000	5% BSA	Cell Signaling Technology	2683
pJNK(Thr183/Tyr185)	Mouse Monoclonal	1:500	5% Milk	Santa Cruz Biotechnology	sc-6254
JNK	Mouse Monoclonal	1:500	5% Milk	Santa Cruz Biotechnology	sc-7345
pIKKβ (Ser177/181)	Rabbit Polyclonal	1:1000	5% BSA	Cell Signaling Technology	2694
IKKβ	Rabbit Polyclonal	1:1000	5% BSA	Cell Signaling Technology	2678
pIRS1 (ser302)	Mouse Monoclonal	1:1000	5% Milk	Millipore	05-1086
IRS1	Rabbit Monoclonal	1:1000	5% BSA	Cell Signaling Technology	2382
Anti-Rabbit Secondary	Mouse Monoclonal	1:10,000	5% BSA or Milk	Cell Signaling Technology	7074
Anti-Mouse Secondary	Donkey	1:5000	5% BSA or Milk	Cell Signaling Technology	7076
